# SERUM PTAU181/NEUROFILAMENT LIGHT ELEVATIONS CORRELATE WITH OPIOID USE AND FECUNDITY IN A PSYCHIATRIC CLINIC

**DOI:** 10.1093/geroni/igae098.3712

**Published:** 2024-12-31

**Authors:** Emily Hanson, Karl Berner, Jon Berner

**Affiliations:** Woodinville Psychiatric Associates, Federal Way, Washington, United States; CONQUER Clinics LLC, Monroe, Washington, United States; Woodinville Psychiatric Associates, Woodinville, Washington, United States

## Abstract

Chart review of initial N=73 cohort screening for dementia risk revealed expected positive control with age across standard ATN measures. All ATN measures were independent of insulin sensitivity (insulin/BMI) Amyloid ratio was uncorrelated with ptau181/Nfl. Broad chart review revealed a divergence between amyloid and tau serum markers: tau associates with opioid use (r. 259, p. 01) and increased fecundity (r. 215, p. 079) while adverse amyloid ratio associates with decreased fecundity (r. -0.223, p.067) and absent statistical association with opioid use. This data is consistent with previous CSF studies documenting elevated ptau181 and TNFa in patients with pain. These results have implications for patient stratification and treatment selection. Granular psychosexual histories may provide an assay for the biochemical tradeoffs associated with harem vs. monogamous mating strategies, obviously implicated the current size disparities in human males vs. females. Ptau 181 elevations associated with a pain behavioral phenotype and CSF elevations of TNFa direct attention to prophylactic medications with known efficacy in human neurogenic pain likely associated with equilibrium distributions of the “M1-like” and “M2-like” microglia: ketamine, rapamycin, and dimethylfumarate. Acute cortisol challenge may also provide useful clinical information regarding the potentially post- translational equilibrium distribution, opaque to RNAseek in autopsy specimens, of the “M2c- like” microglia vs. the IL-4 dependent “M2a-like” microglia.

